# Evaluation of Consecutive Guided Training to Improve Interrater Agreement in Identifying Elements of Situation Awareness in Objective Structured Clinical Examination Assessments

**DOI:** 10.15694/mep.2021.000106.1

**Published:** 2021-04-30

**Authors:** Markus Fischer, Marlies P Schijven, Kieran M Kennedy, Steven Durning, Thomas JB Kropmans

**Affiliations:** 1 School of Medicine; 2 Department of Surgery; 3 Department of Medicine

**Keywords:** Situation Awareness, Undergraduate Medical Education, Objective Structured Clinical Examination, Interrater Agreement, Generalisability Theory

## Abstract

This article was migrated. The article was marked as recommended.

**Introduction:** Little is known about the medical student’s cognitive ability in diagnostic and therapeutic accuracy. Literature does not suggest a methodology to quantify students' cognitive processing. Situation Awareness (SA) is described as having the proficiency to obtain awareness of the surrounding and to integrate this consciousness into the situational context and potential forthcoming development. OSCEs might be a suitable instrument to evaluate students’ awareness of the situation.

**Methods:** Consecutive guided training was provided to obtain a consistent comprehension of the model of SA. 4 independent researchers consecutively examined 6 randomised OSCE forms in a qualitative and quantitative method. Final interrater agreement was expressed as Cohens kappa. Generalisability theory determined the impact of the main facets on the variation in disagreement.

**Results:** Evaluation of identifying and categorising elements of SA within OSCE forms demonstrated a moderate to very good interrater agreement. The G-Theory revealed key facets for variance: OSCE forms, Levels of SA, Items embedded in the Levels, Interaction between Forms and Levels and Forms and Items embedded within Levels.

**Conclusion:** Consecutive guided training improved the identification of elements of SA within OSCE assessments. Further research is necessary to improve the assessment of SA in undergraduate medical curricula.

## Introduction

Medical education aims to qualify physicians in both diagnostic accuracy and subsequently selecting the best treatment option for any given patient presentation ( Wallace, 1997). Clinical Reasoning (CR) includes the fundamental cognitive information processing to reach diagnostic and therapeutic decisions and has been shown to be governed by both the patient’s signs and symptoms and the situation in which they occur. Acquiring CR presupposes both the ability to identify the underlying causes for a patient’s condition as well as the ability to extract and integrate additional information needed to fully understand the clinical situation ( Chamberland *et al.,* 2015). Deficient information processing of physicians is reported throughout the literature, suggesting the exigency to develop strategies to foster more competent cognitive reasoning abilities ( La Pietra *et al.,* 2005; Graber, Wachter and Cassel, 2012). Ongoing research in the field of diagnostic reasoning and clinical errors is mainly carried out retrospectively. To date, not much evidence exists on the issue in professional healthcare settings such as primary care or speciality training ( Singh *et al.,* 2017). Nevertheless, findings within these clinical areas support the identification of subdomains within the CR process that contribute to erroneous consequences. Conclusions of these studies are pointing towards the necessity to develop methods to assess the clinicians’ cognitive ability for diagnostic reasoning ( Cutrer, Sullivan and Fleming, 2013; Zwaan, Schiff and Singh, 2013; Wood, 2014). Furthermore, research outcomes direct the focus on the development of educational strategies which can be implemented into early medical training including assessments such as the Objective Structured Clinical Examination (OSCE) ( Graber, 2009).

The OSCE is a modern type of examination ( Harden *et al.,* 1975) often used to assess performance in the medical domain ( Schijven *et al.,* 2010). It is designed to test clinical skill performance and competence across multiple domains including communication, clinical examination and interpretation of results ( Ross *et al.,* 1988). Derived from the field of aviation, situation awareness (SA) is described as having the proficiency to obtain and maintain consciousness of all particulars in the surrounding and to concurrently integrate the understanding of the situation and the projection of its potential forthcoming development ( Endsley, 2000). Endsley, in her model of SA for high-risk environments accentuated three interdependent levels essential to obtain and maintain awareness of the given situation and to project its possible development in the near future ( Endsley, 1999). It is a model shown fit to describe the dynamic process of receiving, interpreting and processing information in dynamic environments such as the medical field ( Graafland, Bemelman and Schijven, 2015; Graafland and Schijven, 2015). In healthcare, inadequate SA was identified as a primary parameter associated with deficient clinical performance, recommending the implementation of SA training including simulation into medical undergraduate education as realised in other high-risk environments ( Parush *et al.,* 2011; Graafland *et al.,* 2015). The WHO emphasised in 2009 the importance of early exposure of undergraduate medical students to elements of information processing to obtain as well as maintain SA ( WHO, 2009; Walton *et al.,* 2010). The recent implementation of mnemonics such as ISBAR (Identify, Situation, Background, Assessment and Recommendation) or I-PASS (Illness severity, Patient summary, Action list, Situation awareness & contingency and Synthesis by receiver) into healthcare highlights the importance of SA in improving safe and complete transfer of critical information ( Cornell *et al.,* 2014; Starmer *et al.,* 2012). Learners seeking assistance from clinical experts are expected to provide appropriate and pivotal clinical information and observations based on the given presentation of the patient. On the basis of Endsley’s model, elements of medical practice can be assigned to each of the three levels of SA Table 1.

**Table 1.** Elements of clinical practice categorised to the individual Levels of SA based on Endleys’ model

*Level 1 SA*	*Perception of situational elements*
Overall general impression	Diagnostic impression based on the clinical appearance of the patient Environmental scan including items indicating medical impairment of the patient
History taking	Chief complaint or reason for consultation Patients history of course of disease Treatment and drug/ medication history
Physical examination	General physical condition and specific discomfort
Diagnostic test results	Results from common and organ specific diagnostic laboratory tests Findings from diagnostic imaging
***Level 2 SA***	***Comprehension of elements in situation***
Pattern recognition	Recognition of concurrence of clinical signs, symptoms and/ or complaints
Detection of abnormalities	Recognition of unusual and/ or unsuspected findings and pathological changes Identification of information conflict and possible misinterpretation of complaints, signs and symptoms
Formulating working diagnosis	Determination of the most favourable disease based on the clinical presentation and gathered information
Consideration of differential diagnosis	Incorporating all gathered information into critical consideration of optional matching diseases
***Level 3 SA***	***Projection of their meaning for future situation***
Consideration of treatment options	Availability and restrictions of treatment options Harmonisation of the patients’and physicians’preferences
Identification of need for further investigations	Necessity, reliability and validity of additional examinations and tests
Consideration of optional outcomes	Potential consequences (benefit and harm) Harm of optional therapies and additional examinations and tests
Search for expedient additional information	Identification of absence of potentially valuable information Outlook for the patient

The shift from time-based education to competency-based training in medicine necessitates the development of adequate assessment methods ( Gruppen *et al.,* 2017). History taking and physical examination are core skills demonstrated by medical students. However, the ability to integrate the gathered information into further processing steps is a fundamental requirement for CR ( Carraccio and Englander, 2013; Daley and Torre, 2010). Assessment in undergraduate medical curricula rarely incorporate cognitive information processing indicating the development and utilisation of SA embedded in the underlying CR process. Furthermore, evaluating professional skills based on human judgment of behavioural markers of testees are prone to subjectivity of the raters ( Ginsburg *et al.,* 2010). Attempts to mitigate this individual impact were strengthened by standardising the assessment and how the level of performance can be determined ( Van der Vleuten, Norman and De Graaff, 1991).

OSCEs are, in theory, intended to assess student's competencies under variable circumstances ( Zartman *et al.,* 2002). Whole-task OSCEs including elements of all three levels of SA have been suggested to inspire students to develop cognitive abilities to obtain diagnostic and therapeutic accuracy ( Durak *et al.,* 2007). OSCEs do not provide a comprehensive evaluation of an overall competency ( Lurie, Mooney and Lyness, 2009). However, if set up as summative evaluation, this type of assessment is suggested to draw an informative compilation of the students’ ability to integrate various competencies ( Gormley, 2011; Van der Vleuten and Schuwirth, 2005; Wass *et al.,* 2001). Thus, OSCEs might be a suitable instrument to evaluate students’ understanding of the situation as part of their CR and subsequently to provide deductive feedback of their cognitive processing upon completion of the patient encounter ( Fischer *et al.,* 2017). However, literature does not suggest an accepted methodology to quantify students' utilisation of SA in a clinical encounter. Simply assuming that accurate SA automatically matches reasonable performance and vice versa has been disproven ( Endsley, 2000), raising the question as to whether elements of SA could be taught and assessed in undergraduate medical education. In a preliminary study we evaluated a self-developed assessment tool for its validity and interrater reliability for identifying elements of SA within freely available OSCE guides and OSCE score sheets utilised in medical education ( Frere *et al.,* 2017). Upon an initial introduction to the model of SA and its adaption to healthcare, raters were able to identify elements of SA, however, only moderate interrater reliability has been demonstrated. The classical psychometric analysis does not provide insight into the causes of this level of disagreement as the percentage of agreement is not corrected for change and therefore, this correction is random. Generalizability Theory (GT) analysis supports the identification of the variability of sources of error around the observed score of agreement ( Shavelson, Webb and Rowley, 1989). GT consists of a Generalisability-Study (G-Study) and a Decision-Study (D-Study). In the G-Study the main facets of variation and all their interactions are being examined. The D-Study allows to calculate the effect of experimental measurement designs on the reduction of the error around the observed score ( Trejo-Mejía *et al.,* 2016). The Standard Error of Measurement (SEM) is described as the error around the observed score and is expressed in the same unit of measurement as the assessment tool used (% agreement). An observed score is the result of the unknown true score and error around the observed score. The true score is an optimal score out of the universe of potential scores ( Bloch and Norman, 2012).

The purpose of this study is 1. to evaluate the effect of consecutive ‘guided’ training on the improvement of interrater agreement, 2. to assess the reliability of a method for identifying elements of SA embedded in Objective Structured Clinical Examination (OSCE) station score sheets, which can be categorised to the three levels of SA (Level 1 SA, Level 2 SA, Level 3 SA) based on Endlesy’s model, and 3. to identify facets contributing to interrater disagreement.

## Methods

Four independent researchers (1 primary investigator, 1 senior lecturer in Medical Informatics & Education, 1 medical practitionerand 1 allied health care practitioner) consecutively examined 6 randomised OSCE guides/ score sheets in a qualitative and quantitative method. This mixed method utilised a self-developed node tree using NVIVO 10 software, allowing for coding of information to predefined nodes by multiple researchers and subsequent interrogation of diverse types of queries and comparisons (NVIVO version 10.0.638.0 SP6 32-bit). Interrater agreement (Cohen’s Kappa) was calculated by NVIVO based on levels of agreement (%) and disagreement (%) ( Bazeley and Jackson, 2013). Interrater agreement was considered as very good if Cohen’s Kappa is > 0.80, good when ranging between 0.60 - 0.80 and moderate when ranging between 0.40 - 0.59. To analyse the impact of the main facets on the variation between Levels of SA (L), the Items (I) embedded within these levels and the 4 Raters (R), we utilised EduG for the G-study and G-study analysis ( Cardinet, Johnson and Pini, 2011). Generalisability Coefficient (G-coefficient) is considered as the reliability coefficient addressing agreement between examiners. The main facets of analysis were defined by the Analysis of Variance (ANOVA) as OSCE Forms (F), Individual Raters (R) and the 3 Levels of SA (L). Two consecutive designs of measurement were chosen to 1. Analyse the Raters as object of measurement (R/FLI) and 2. Analyse the OSCE scoring sheets as object of measurement (F/RLI).

**Figure 1.**
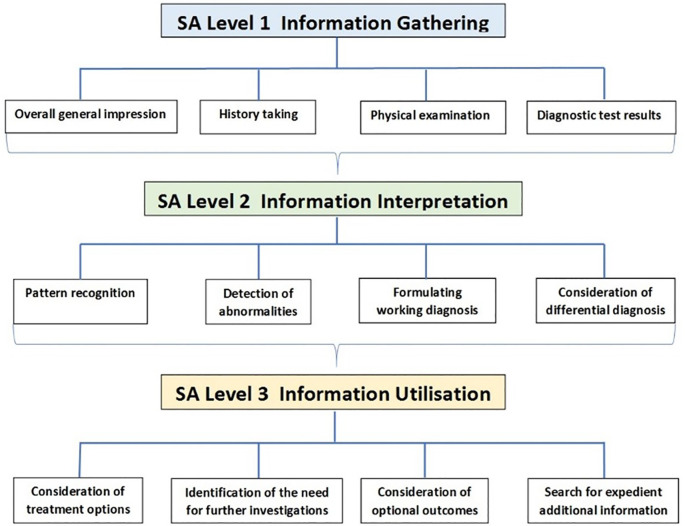
Node Tree developed for coding of elements of Situation Awareness

Initially, a phased course of action was developed. In phase 1, elements extracted from papers identifying underlying causes of diagnostic and treatment errors in clinical practice were classified to the individual levels of SA in our model used. Subsequently, this information was critically appraised and elements categorised to facets. In phase 2, an assessment tool was developed based on the collected data in phase 1, aiming to enable educators to evaluate elements of SA. This tool was subsequently utilised to develop the node tree utilised for coding of OSCE forms with NVIVO ( Figure 1). Essential steps during a patient encounter were identified and incorporated into the node tree.

### Consecutive Guided Training

**Figure 2.**
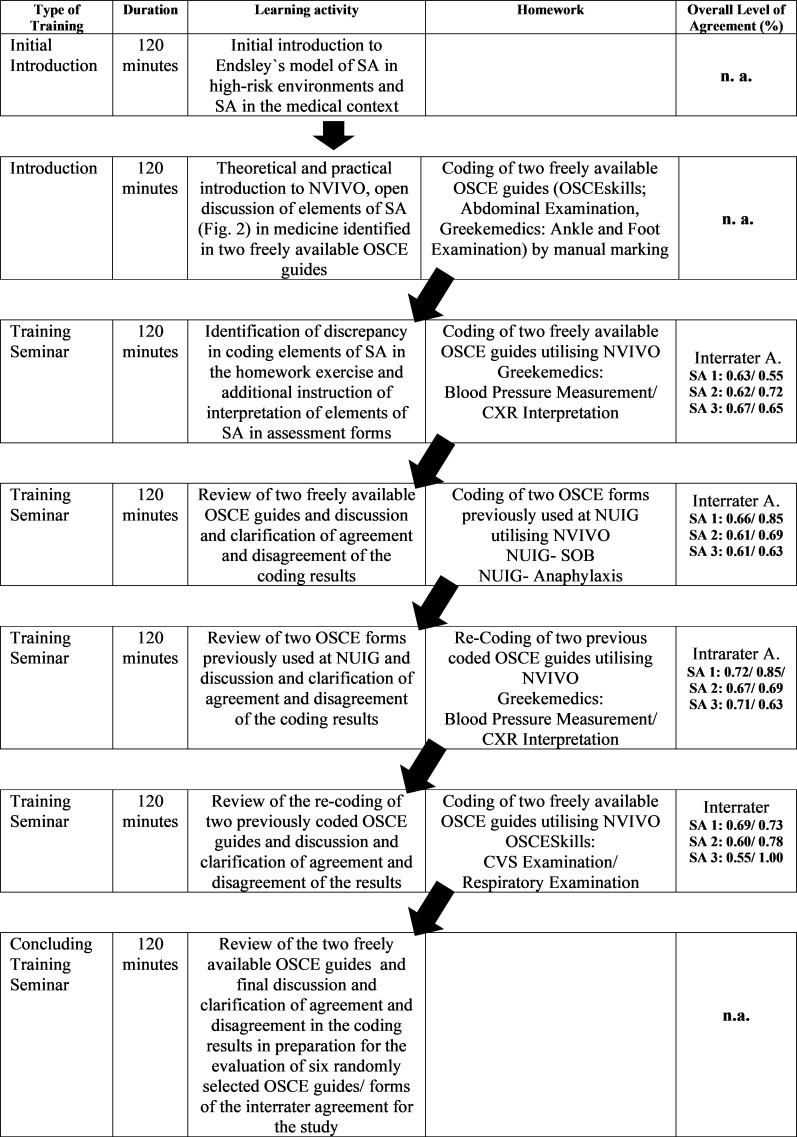
Structure of the Training of the Researchers and Evaluation of Coding Elements of SA within OSCE guides and OSCE score sheets

An introduction session was provided initially to research fellows to obtain a consistent comprehension of the model of SA by Endsley and characteristics attributed to SA in the medical context ( Table 1). Subsequently, two freely available OSCE guides potentially preparing students for their evaluation of clinical skills (( https://osceskills.com, 2017) now https://www.medistudents.com/osce-skills and https://geekymedics.com/category/osce/; both accessed on 01. 04. 2017) were analysed and discussed openly utilising the self-developed node tree ( Figure 1). Elements of the clinical assessment had to be appraised for the Level of SA, and subsequently assigned to the most appropriate facet within this level. In case of identification of diverging elements of SA within one phrase, the text passage had to be split and coded individually to the selected facet. Headlines, description of images, expected learning outcomes or educational instructions within the OSCE forms were excluded form coding. This was followed by the evaluation of further two freely available OSCE guides as individual home exercise. Upon appraisal of the interrater disagreement identified in the home exercise, the researchers openly discussed any discrepancy to achieve optimal understanding of the meaning of parental nodes and child nodes. This was followed by successive evaluations of further four homework exercises including two randomly selected freely available OSCE guides and OSCE score sheets utilised at the National University Ireland Galway (NUIG) respectively at any one time. All 8 forms were independent from the actual study. Progress in the level of interrater agreement was determined by calculating Cohen’s Kappa. Ongoing disagreement was openly discussed to achieve concordance and final decisions were adjudicated by the principal investigator if deemed necessary. Upon proven increment in agreement between researchers three randomly selected freely available OSCE guides and three randomly selected OSCE forms of the medical training at NUIG (2015-2016) were analysed individually for the final study ( Figure 2).

## Results

**Figure 3.**
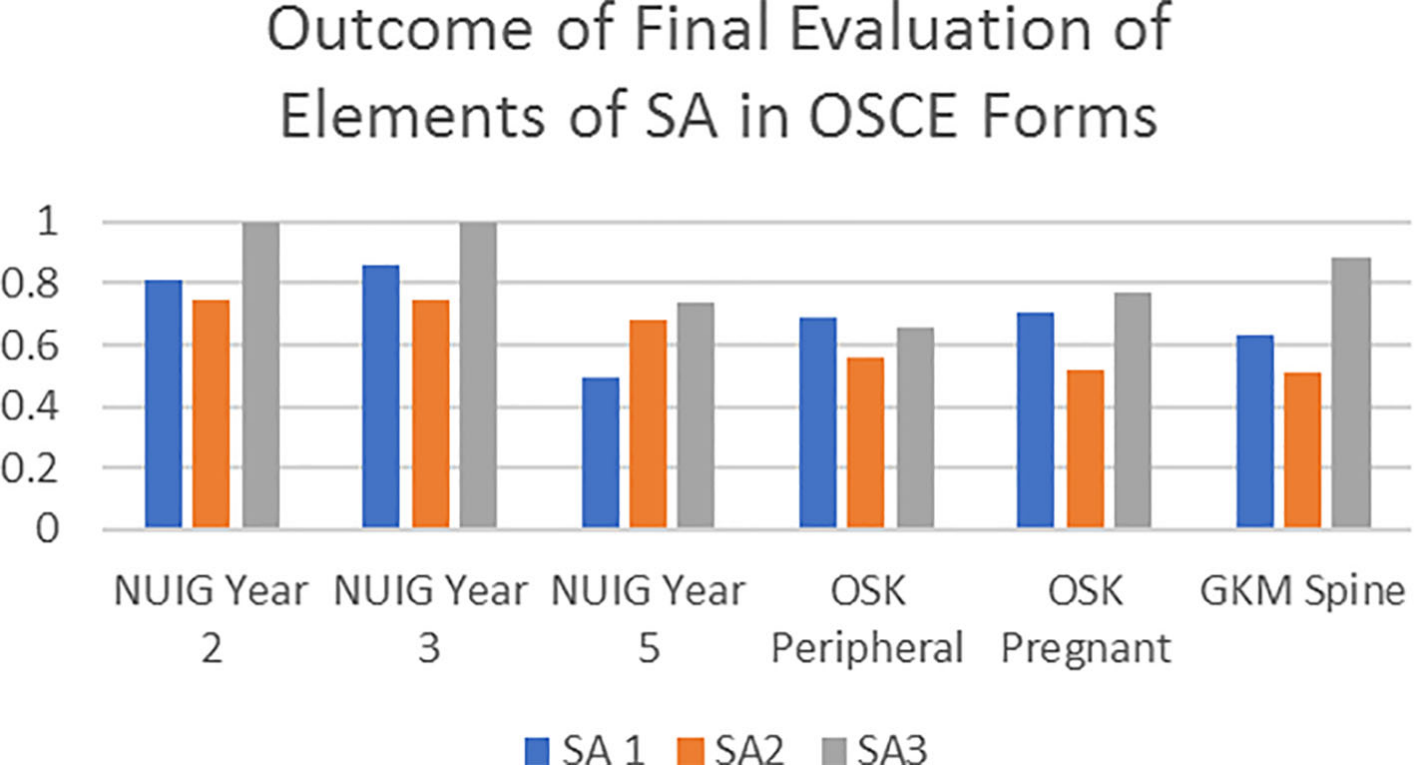
Overall interrater agreement (classical psychometric analysis) of levels of SA within six OSCE forms between 4 independent raters

Identifying and categorising elements of SA within OSCE forms by 4 individual researchers demonstrated a moderate to very good interrater agreement based on Cohen’s Kappa (0.497 - 1.00) ( Figure 3). The G-Theory revealed four key facets for variance: OSCE Forms/Scoresheet (F) (n=6); the Independent Raters (R) (n=4); the Levels of Situational Awareness (L) (n=3) and the Items embedded in these Levels of SA (I:L) (n=5). The absolute G-coefficient of the reliability study was 0.92 as compared to the results of the classical psychometric analysis.

Of all variance, 2.7% is due to the OSCE score sheets, 0.4% is due to individual raters. 8.9 % of variance can be attributed to the distinct Levels of SA. Most of the main facets responsible for the variance were associated with the ‘Items embedded in the Levels’ seen as high as 32.7 %. Furthermore, 0.6 % of variance is due to the effect of the interaction between Forms and Raters (raters being influenced by the different types of forms), 15.2 % are associated with interaction between Forms and Levels and 20.3 % with Forms and Items embedded within Levels. Additionally, small interaction effects were identified with a residual unexplained error of 9.3 % ( Table 2).

**Table 2.** Analysis of variance including all facets and experimental interdependency

Source	SS	df	MS	Random	Mixed	Corrected	%	SE
F	125.02222	5	25.00444	-0.35422	0.39407	0.39407	2.7	0.40062
R	18.26667	3	6.08889	-0.06252	0.05254	0.05254	0.4	0.09116
L	589.08889	2	294.54444	0.75707	1.96104	1.30736	8.9	1.81590
I:L	1917.95000	12	159.82917	6.01981	6.01981	4.81585	32.7	2.51926
FR	20.40000	15	1.36000	-0.17378	0.09067	0.09067	0.6	0.07306
FL	488.64444	10	48.86444	1.64765	2.24489	2.24489	15.2	1.00584
FI:L	799.25000	60	13.32083	2.98620	2.98620	2.98620	20.3	0.59921
RL	85.93333	6	14.32222	0.27743	0.34519	0.34519	2.3	0.24244
RI:L	122.71667	36	3.40880	0.33880	0.33880	0.33880	2.3	0.13254
FRL	119.00000	30	3.96667	0.51813	0.79333	0.79333	5.4	0.20042
FRI:L	247.68333	180	1.37602	1.37602	1.37602	1.37602	9.3	0.14425

### Raters as Object of Measurement

Using a measurement design in which the main sources of variation are the raters it appeared that 81.3 % of the variation is due to the forms whereas 18.7 % is due to the raters with an overall G-coefficient of 0.39. An assumptive increase in the amount of forms being analysed suggested an increment of the reliability with the G-coefficient raising from 0.39 to 0.65. The SEM can experimentally be reduced from 28% to 14 % when quadrupling the number of OSCE forms to be analysed. The G-facets analysis based on raters as object of measurement demonstrated the level of unreliability for each individual OSCE form. In this measurement setting, the absolute G-coefficient for the 6 individual forms utilised in the final assessment ranges from 0.208 to 0.487, indicating low reliability of the OSCE forms. The G-coefficient for the individual levels SA 1, SA 2, SA 3 was calculated as 0.296, 0, 0.032 and 0.000 respectively, indicating a poor reliability of these facets.

### OSCE Forms as Object of Measurements

The measurement design analysing the impact of the OSCE forms (F/RLI) revealed that 36.7 % of variance was related to the raters and 63.3 % of variance was assigned to the forms being analysed. The optimisation using a D-study revealed that an increase in the number of raters (from 4 to 6, 8 and 10) analysing a fixed number of forms (6) only contributes to an increase of about 4 % in reliability. The associated Standard Error of Measurement would improve from 19% (0.189) to 12% (0.119). The G-coefficient, indicating the reliability of each of the 4 raters varies between 0.84 to 0.96, suggesting an overall high reliability. The results for the individual Levels of SA demonstrate Level 1 to be very reliable, Level 2 as not reliable and Level 3 as less reliable.

## Discussion

To our best knowledge, this was the first study to evaluate the impact of a consecutive guided training on how different raters are able to identify levels of SA. The outcome suggests that providing training enables educators and examiners to understand the concept of SA and to identify elements of SA within medical performance and competency assessments. That goes for each level of SA based on Endsley’s model being perception of situational elements, comprehension of elements in situation and the projection of their meaning for future development of that situation ( Graafland *et al.,* 2015). All OSCE score sheets used in our samples were designed without incorporating any specific knowledge or training in SA. We picked a random selection of forms of freely available OSCE guides and OSCE score sheets from a single medical curriculum of which no evidence showed that SA was part of the curriculum e.g. part of the assessment. However, the results of our study revealed that the OSCE Forms and the Items embedded in the individual Levels of SA are not reliable for the purpose of assessment of SA. The results for the individual Levels of SA demonstrated Level 1 to be very reliable, Level 2 as not reliable and Level 3 as less reliable. The low occurrence of elements which can be attributed to the Levels 2 SA and 3 SA within the 6 OSCE forms utilised for the study might be causative for the poor outcome. Compared with the outcome of the preliminary study ( Frere *et al.,* 2017), Cohen’s Kappa in our evaluation demonstrated an improved outcome of interrater agreement, ranging between moderate and very good levels of agreement. This suggests that the consecutive guided training provided to researchers had beneficial impact. The G-theory revealed no significant improvement of the results by the addition of further raters(G-coefficient raised from 0.92 to 0.96 when doubling the number of raters). In contrast, the addition of OSCE guides and score sheets did show an improvement of interrater agreement (from 0.39 to 0.65 when quadrupling the number of OSCE Forms). The amendment of the SA score description was identified as one key contributor to a superior outcome. A clear instructional outline of the expected activities and behaviour is suggested to support the intelligibility of OSCE score sheets by individual raters, thereby fostering the standardisation of the qualitative and quantitative evaluation of cognitive processing within clinical competence examinations.

Assessment of the development of clinical expertise remains a challenge to medical education as cognitive performance cannot be evaluated by direct observation ( Huwendiek *et al.,* 2010). Furthermore, fundamental cognitive processes in developing clinical expertise by medical students have not been clearly identified. This results in a lack of instructional measures enabling the development of the cognitive competence as part of CR in medical students ( Kiesewetter *et al.,* 2013). The necessity of the ability to categorise assessment criteria of the OSCE score sheets into the elements amongst each level of SA attenuates the need for training of medical examiners. Rater-based assessments have been identified as possibly biased and interrater reliability as poor ( Carline *et al.,* 1992). Raters are influenced by own cognitive and perceptual abilities and limitations when assessing testees which might impact the quality of their judgment of students’ performance ( Downing, 2004). This highlights the need for assessors to be able to adequately identify cognitive abilities as one cornerstone of clinical competence. Based on research outcomes in underlying science of diagnostic errors, Singh and Sittig recommended the reconfiguration of training and education as well as the development of assessment methods to measure the quality of diagnostic care ( Singh and Sittig, 2015). An analytical tool to identify breakdowns in SA in the underlying diagnostic process could differentiate elements within the clinical encounter which can be categorised into the level of SA ( Singh *et al.,* 2012). Whole-task OSCEs are suggested to enable the evaluation of the utilisation of SA ( Fischer *et al.,* 2017). Fida and Kassab indicated that scores achieved by medical students in OSCE stations strongly correlated with the students' ability to select and incorporate pertinent information and competence in patient management ( Fida and Kassab, 2015). The summative evaluation of the integration of various competencies by individual assessors might facilitate a scoring system enabling the inferring of the underlying cognitive process of medical students ( Lomis *et al.,* 2017). For example, the satisfactory completion of a thoroughly history taking or physical examination by the student suggests an adequate Level 1 SA. Subsequently formulating of an incongruously working diagnosis, however, might suggest a flawed incorporation of the gathered information in subsequent cognitive processing correlating with deficiencies in Level 2 SA. Wilkinson *et al.* demonstrated a correlation between direct involvement of the examiners in the designing of OSCEs and interrater reliability. Collaboration in the development of clinical assessment stations including objectives, format and score sheets were suggested to improve subsequent examiners understanding ( Wilkinson *et al.,* 2003). However, clinicians who developed their expertise over many years are commonly unaware of the levels of SA and, thus, they generally cannot convey or teach this process of data gathering and incorporation into the judgmental process ( Ilgen *et al.,* 2012). Our study demonstrated that clinical practitioners and medical educators can be trained in understanding of the meaning of elements of SA in the medical context identified in assessment forms. Though, identifying of key elements of cognitive competencies within medical assessments were demonstrated as being difficult.

## Conclusion

Situation Awareness (SA) is vital skill for today’s healthcare professional, at the same time it is a difficult concept to measure validly. While training and assessment are increasingly incorporated into medical practice, evidence on how to train and assess SA in medical education best is largely lacking. Our study shows that elements identified in OSCE score sheets can be attributed to individual levels of SA. Such forms may help in enabling the development of a cognitive map giving insights in information processing among medical students. Thus, deficits in recognising and incorporating essential parameters during assessment can be identified and remediated when developing clinical expertise. This potentially can prevent the necessity of tackling habits already evolved over time. Further research is necessary to improve the assessment of SA and to determine to which degree OSCE assessment forms can be utilised to identify where the chain of SA was broken down. Ultimately, it may help in facilitate the development of educational strategies fostering cognitive reasoning abilities among medical students.

## Take Home Messages

• Patient Safety raised increasing public attention over the last years, often due to diagnostic or treatment errors including prescribing wrong mediations.
• Situational awareness (SA) is one key element of the so-called non-technical skills and the essential prerequisite for subsequent diagnostic and clinical reasoning.
• Studies suggest that students have little insight into cognitive reasoning in clinical scenarios.
• Clinical practitioners and medical educators can be trained in understanding of the meaning of elements of SA in the medical context identified in assessment forms.
• Though, identifying of key elements of cognitive competencies within medical assessments were demonstrated as being difficult.

## Notes On Contributors

**Markus Fischer** was responsible for the project and study design and was the primary author. Markus A Fischer is an allied healthcare professional. He completed his a PhD in the field of Situation Awareness in Medical Education at the School of Medicine, National University Ireland, Galway. His research interest is with Human Factors in Clinical Healthcare Areas and Medical Education.

**Marlies P Schijven** was co-author of the paper and involved in providing guidance in the application of analysis of Situation Awareness in Medicine. Marlies P Schijven, MD PhD MHSc is a Professor and a Surgeon at the Academic Medical Center Amsterdam, The Netherlands, Previous President of the Dutch Society of Simulation in Healthcare. M.P. Schijven is one of the AMC Principal Investigators. Her focus of research: Simulation, Serious Gaming, Applied Mobile Healthcare, Virtual Reality Simulation. ORCiD Link: https://orcid.org/0000-0001-7013-0116

**Kieran M Kennedy** contributed to the interpretation of the data and revising the manuscript. Kieran M Kennedy is a Lecturer in Clinical Methods and Clinical Practice in the School of Medicine at the National University of Ireland Galway. He is involved in teaching undergraduate medical students at all stages of their training as well as the development of OSCE stations and OSCE score sheets. ORCiD Link: https://orcid.org/0000-0002-9481-5424

**Steven Durning** was involved in interpreting the data and revising the manuscript. Steven Durning is a Professor of Medicine and Pathology at the Uniformed Services University (USU) and is the Director of Graduate Programs in Health Professions Education. As an educator, he mentors graduate students and faculty, teaches in the HPE program and directs a second-year medical school course on clinical reasoning. As a researcher, his interests include enhancing our understanding of clinical reasoning and its assessment. ORCiD Link: https://orcid.org/0000-0001-5223-1597

**Thomas JB Kropmans** contributed to the design of the study, analysis and interpretation of the data, drafting and revising the manuscript. Thomas JB Kropmans is a Senior Lecturer in Medical Informatics and Medical Education in the School of Medicine at the National University of Ireland Galway. His research interests include postgraduate medical education and continuing professional development. ORCiD Link: https://orcid.org/0000-0001-7071-3266
