# Supplementary material for: Evaluation of Consecutive Guided Training to Improve Interrater Agreement in Identifying Elements of Situation Awareness in Objective Structured Clinical Examination Assessments
Source: MedEdPublish (2016). 2021 Apr 30;10:106. [Version 1] doi: 10.15694/mep.2021.000106.1 (PMC10939566; doi:10.15694/mep.2021.000106.1)
Supplement: Peer review history

# Reviewer report: Reviewer response for version 1

**DOI:** 10.21956/mep.19098.r27529

**Published:** 2021-08-04

**Authors:** Julie Hunt

**Affiliations:** 1 Lincoln Memorial University

This review has been migrated. The reviewer awarded 4 stars out of 5 This paper describes the assessment of situational awareness (SA) in OSCEs and focuses on the measurement of inter-rater agreement, as measured by cohen’s kappa and a generalizability study. The authors make a good case in the introduction and discussion that SA is worth assessing in students, and their results suggest that when that SA is assessed using items on an OSCE rubric, that most of the variance can be attributed to the rubric items that are embedded in the levels, and with forms and items embedded within levels--not with the raters. I struggled to follow how they used an “assumptive increase in amount of forms” to adjust the G-coefficient; I would have appreciated more explanation of that conversion.The authors describe a consecutive guided training for raters, and repeating their results (mostly acceptable inter-rater reliability) would be dependent on adequate/significant rater training. This paper serves as a proof-of-concept study and suggests that SA can be evaluated with adequate reliability in an OSCE format.

Reviewer Expertise:

NA

No decision status is available

# Reviewer report: Reviewer response for version 1

**DOI:** 10.21956/mep.19098.r27530

**Published:** 2021-05-27

**Authors:** Rukhsana Zuberi

**Affiliations:** 1 Faculty of Health Sciences, Aga Khan University

This review has been migrated. The reviewer awarded 3 stars out of 5 The paper was an excellent read. The number of forms and OSCE Score Sheets assessed were few. Three improving comments: 1. If more score sheets or OSCE guides had been analysed the authors may have obtained much better result.2. An additional D-Study may have provided additional results: fewer Raters and More numbers of OSCE forms.3. There was a concern that although Kappa is good for interrater agreement beyond chance, it is better for categorical items. Interrater Reliability with ICC may have been better here.Overall: Excellent effort. I enjoyed reading it.

Reviewer Expertise:

NA

No decision status is available
